# Anaphylaxis caused by flaxseed

**DOI:** 10.1186/2045-7022-5-S3-P58

**Published:** 2015-03-30

**Authors:** Ramon Lleonart, Blanca Andrès, Fernando Pineda, Moises Labrador, Mercè Corominas

**Affiliations:** 1 Allergy Section, Hospital Universitari de Bellvitge, Barcelona, Spain; 2 Laboratorio Diater, Madrid, Spain; 3 Allergy Section, Hospital Universitari Vall d’Hebron, Barcelona, Spain

## Background

Flaxseeds are the seeds of the plant *Linum usitatissium* belonging to the *Linaceae family*. They are appreciated for its antioxidant properties and may be add to different foods.

## Case report

A 44-year-old man presented with dyspnoea and generalized urticaria after the ingestion of a yogurt with flaxseed. Clinical history revealed an anaphylactic reaction after nuts consumption and an oral allergy syndrome after peach ingestion. He had a history of moderate allergic rhinoconjunctivitis and mild intermittent asthma.

## Methods and results

Prick-prick carried out with flaxseed showed a strong positivity. Skin prick test to inhaled allergens were positive to mites, dog and cat danders, plain tree and grasses. Skin prick test to food allergens were positive to almond, hazelnut, peanut, chestnut, walnut, mustard, lentils, soybeans, rice, oat, corn and lupine. Total IgE was: 291 UI/ml. Specific IgE (CAP, ThermoFisher Scientific) to flaxseed was 2.68 kU peach 7.65 kU/L, peanut 2.01 kU / L , lettuce 0.92 kU / L , lupin 3.76 kU / L. Corn IgE 2.71 kU / L , Pru p3 7.62 kU / L , Cor a8 0.68 kU / L. ISAC, ImmunoCAP was positive to Fel d1, Phl p1, Der f1, Der f2, Der p1, Der p2, Pla a3, Ara h9, Cor a8, Jug r3, Pru p3 and Art v3. ISAC performed after inhibit patients's serum (ISAC inhibition) with a complete flaxseed extract (f233:Linum usitatissium from ImmunoCAP, ThermoFisher Scientific) did not reduced the positivity to non-specific lipid transfer proteins (nsLTP). ISAC inhibition with a complete mite extract (d1: D. pteronyssinus from ImmunoCAP, ThermoFisher Scientific) reduces exclusively the signal of mites (Der f1, Der f2, Der p1, Der p2) in >83%.An immunoblot performed with flaxseed extracts (soluble and liposoluble), showed the presence of an IgE-binding band of about 18 kDa in the liposoluble fraction and a 20 kDa band in the soluble one.

## Conclusion

We present a case of anaphylaxis after eating a yogurt containing flaxseed, in a patient polysensitized to inhaled allergens and nsLTP. IgE-mediated hypersensitivity has been demonstrated by *invivo* and *in vitro* tests. The ISAC inhibition rejects the nsLTP as the allergen responsible of this hypersensitivity, the allergen of 18 KDa detected in the liposoluble fraction of flaxseed could correspond to an oleosin. Flaxseed allergy has rarely been reported and to the best of our knowledge this is the first report where an oleosin could be the responsible of flaxseed allergy.

## Consent

Written informed consent was obtained from the patient for publication of this abstract and any accompanying images. A copy of the written consent is available for review by the Editor of this journal.

